# Outcomes and management of de novo ureteral strictures following ureteroscopy: A retrospective observational study

**DOI:** 10.1177/03915603261418995

**Published:** 2026-02-08

**Authors:** Anette Nyheim, Øyvind Ulvik, Christian Beisland, Patrick Juliebø-Jones

**Affiliations:** 1 Department of Clinical Medicine, University of Bergen, Norway; 2 Department of Urology, Haukeland University Hospital, Bergen, Norway; 3 The Clinical Research in Aging and NEphro-urology (CRANE) Group, Bergen, Norway

**Keywords:** ureteroscopy, ureter, stricture, iatrogenic, complication

## Abstract

**Introduction:**

Ureteral strictures are a recognised late complication post ureteroscopy (URS). Most research on the pathology focuses on mechanisms of injury and preventative strategies. There is limited data describing follow up patterns and treatment outcomes. The aim was to evaluate the subsequent management and outcomes of de novo ureteral strictures post URS.

**Methods:**

Retrospective review was conducted of patients diagnosed with a de novo ureteral stricture post URS at a tertiary centre between 2014 and 2024. Eligible cases were identified by manual review of ICD-coded records (N13.0–N13.5), yielding 40 patients for analysis. Baseline and procedural data were summarised descriptively, and group comparisons were performed using the Mann–Whitney U and Fisher’s exact tests.

**Results:**

Among 40 patients (median age 69 years), most strictures were detected symptomatically (67.5%) and were located proximally or distally (each 43%). Median time to diagnosis was 3.8 months, and median follow-up was 4.3 years. Overall, 45% achieved successful resolution after a median of two procedures, while 15% underwent nephrectomy. Higher age-adjusted Charlson Comorbidity Index scores and older age were significantly associated with poorer outcomes, although interpretation is limited by the sample size. Stricture length and a history of stone impaction showed no significant associations.

**Conclusions:**

In this cohort, fewer than half of patients with de novo ureteral strictures following URS achieved lasting resolution despite multiple interventions. These findings highlight the potential clinical burden of this complication and support the importance of preventive strategies, timely detection and structured follow-up, while underscoring the need for further studies to better define long-term outcomes.

## Introduction

Urolithiasis is a common condition, estimated to affect up to 12% of individuals during their lifetime.^1^ Globally, its incidence is widely reported to be rising, often attributed to lifestyle factors such as obesity and increased incidental detection on cross-sectional imaging.^2,3^ When surgical intervention is indicated, several alternatives are available including shockwave lithotripsy (SWL), percutaneous nephrolithotomy (PCNL) and ureteroscopy (URS).^4–7^ Robotic pyelolithotomy represents an additional surgical option in selected cases, especially for patients with large stone burdens or challenging renal anatomy.^8–10^ Among these, URS has become an increasingly adopted choice.^11^ For example, in Canada it accounts for 73.5% of all surgeries performed for urolithiasis.^12^ The absolute number of URS procedures is substantial, with over 150,000 carried out annually in the United States alone.^13^ URS can deliver high stone free rates and the morbidity in the early post operative period is relatively low. Late complications can occur however, and this includes development of ureteral stricture(s). Regarding the latter, in a meta-analysis of 43 studies, Moretto et al., reported an overall pooled incidence of 1.9%, increasing to 2.7% in more recent studies and to 4.9% in cases of impacted stones.^14^ With the high number of procedures being performed and the development of new technologies for use during lithotripsy, such as higher-powered laser systems, ureteral stricture formation as a result of URS has become the subject of increasing attention.^15,16^ The focus has generally been on mechanisms of injury, for example, the high temperatures generated by high-watt output, mechanical forces applied during instrumentation and lasering technique.^15,17–20^ In contrast, much fewer studies have documented the subsequent management and outcomes of such strictures after diagnosis.^21^ The aim of this study was to evaluate the subsequent management and outcomes of de novo ureteral strictures diagnosed after URS.

## Methods

A retrospective review was performed of adult patients (⩾18 years) diagnosed with a de novo ureteral stricture following URS. The study was performed at Haukeland University Hospital, a tertiary endourology centre in Western Norway serving a population of approximately 1 million people.

## Patient identification and eligibility criteria

Potential cases were identified through manual review of all electronic records coded with ICD-10 diagnostic codes N13.0–N13.5.

Inclusion criteria:

1. Age ⩾18 years
2. Underwent URS for stone disease
3. Subsequently diagnosed with a new (de novo) ureteral stricture after URS

Exclusion criteria:

1. Pre-existing ureteral strictures
2. Prior ureteral reconstruction
3. Ureteral strictures not temporally related to a preceding URS procedure

A final cohort of 40 patients met these criteria.

## Stricture detection and follow-up

Strictures were identified either when patients presented with symptoms such as flank pain or urinary sepsis, or during scheduled follow-up imaging, or incidentally on radiological studies or at a later URS procedure. Non-contrast CT (NCCT) was the primary initial imaging modality for assessment.

When upper tract dilatation was observed on NCCT, additional diagnostic evaluations were undertaken as clinically indicated. These included contrast-enhanced CT, or diuretic renography to confirm the presence and extent of the ureteral stricture.

## Management approach

All patients underwent diagnostic ureteroscopy with retrograde pyelography, with or without an attempt at balloon dilatation, to confirm the presence and characterise the extent of the stricture. Endoscopic techniques, including balloon dilatation and/or laser incision, were performed as appropriate on a case-by-case basis. These procedures were carried out by attending surgeons with subspecialist expertise in endourology.

If endoscopic management was unsuccessful, cases were discussed collegially with reconstructive urologists to assess suitability for more invasive reconstructive surgery, taking into account factors such as stricture characteristics, the patient’s comorbidity profile, and patient preferences.

Treatment success (‘successful resolution’) was defined as radiological absence of ureteral obstruction on follow-up imaging, without the need for ongoing drainage (nephrostomy or indwelling stent) and without stricture-related symptoms.

## Data collection and statistical analysis

Baseline demographics, stricture characteristics (location and length), imaging findings and details of surgical management were collected. Descriptive statistics were used for baseline variables. Group comparisons were performed using the Mann–Whitney U test for continuous or ordinal variables and Fisher’s exact test for categorical variables. Statistical analyses were performed using IBM SPSS Statistics 29.0. Statistical significance was set at *p* < 0.05.

## Ethical considerations

This study was conducted as a registered clinical audit and, in accordance with local regulations, was exempt from the requirement for research ethics committee approval.

## Results

### Demographics and presentation

Across the study population (*n* = 40), the median age was 69 years (IQR 56–77), with a male-to-female ratio of 5:3. Most strictures were detected after patients developed symptoms (67.5%), while 22.5% were identified on follow-up imaging, 5% incidentally, and 5% during repeat URS. Among symptomatic cases, flank pain was the most common (71%), followed by urinary sepsis (25%) and recurrent stones (4%). Non-contrast computed tomography (CT) was the standard imaging method for detection of de novo stricture(s) in 38 cases (95%; Table 1).

**Table 1.** Baseline data.

Sample size	40
Age (years), median (IQR)	69 (56–77)
Follow up time (months), median (IQR)	49 (19–92)
Male-female ratio	5:3 (25:15)
ACCI, median (ACCI)	4 (2–6)
Living situation	
Independent	33 (82.5%)
Care assistance	6 (15%)
Nursing home resident	1 (2.5%)
Laterality	
Right	12 (30%)
Left	28 (70%)
Presentation	
Symptomatic	27 (67.5%)
Follow up imaging	9 (22.5%)
Incidental	2 (5%)
At repeat URS	2 (5%)
Symptoms at presentation	
Flank pain	17 (71%)
Sepsis	6 (25%)
Recurrent stone	1 (4%)
Imaging type	
CT	38 (95%)
USS	16 (40%)
Location of ureteral stricture	
Proximal	17 (42.5%)
Mid	5 (12.5%)
Distal	17 (42.5%)
Whole length	1 (2.5%)
>2 cm in length	24 (60%)
Impacted stone at time of URS	30 (75%)
Time (months) between last URS and diagnosis, median (IQR)	3.8 (2.5–8.6)
Total number of URS procedures pre diagnosis	1 (1–2)
New onset CKD	10 (25%)
Number of surgeries to repair stricture, median (IQR)	2 (1–3)

### Stricture characteristics

The ureteral strictures were located proximally in 17 patients (42.5%), in the mid-ureter in 5 (12.5%), distally in 17 (42.5%), and along the entire ureter in 1 (2.5%). Twenty-four strictures (60%) were >2 cm in length, and 30 patients (75%) had been recorded to have had an impacted stone at the time of their URS. The median time from the last URS to diagnosis was 3.8 months (IQR 2.5–8.6), and the median number of URS procedures performed before stricture diagnosis was 1 (IQR 1–2). Fourteen patients (35%) developed a functionless kidney (<30% function), and 10 (25%) had developed new-onset chronic kidney disease (CKD). Median follow up time from stricture diagnosis to final follow up available was 4.3 years (IQR = 1.2–7.9).

### Management and outcomes

The median number of surgeries performed in an attempt to repair a stricture was two (IQR 1–3). All patients underwent diagnostic URS with or without balloon dilatation; 5 patients (12.5%) received laser incision and 10 (25%) underwent reconstructive surgery, of which 5 (50%) were successful. Overall, 18 patients (45%) achieved successful resolution. At last follow-up, 18 (42.5%) had complete resolution, 6 (15%) had undergone nephrectomy, 9 (22.5%) required lifelong nephrostomy, 1 (2.5%) required lifelong JJ stenting, and 6 (15%) were managed conservatively without symptoms (Table 2).

**Table 2.** Follow up data.

	n (%)
Type of surgeries performed	
Diagnostic URS +/– balloon dilatation	40 (100%)
Laser incision	5 (12.5%)
Reconstruction (pyeloplasty/resection/re-implantation)	10 (25%)
Successful reconstruction	5 (50%)
Successful resolution overall (all patients)	18 (45%)
Long term status	
Complete resolution	18 (42.5%)
Nephrectomy	6 (15%)
Lifelong nephrostomy	9 (22.5%)
Lifelong JJ stent	1 (2.5%)
Conservative – asymptomatic	6 (15%)

### Predictors of outcome

There was no significant difference in median time from URS to stricture diagnosis between patients who developed new-onset CKD and those who did not (17 vs 19 months, *p* = 0.7). Larger strictures (>2 cm) showed a lower likelihood of overall successful resolution (37.5% vs 56.3%, *p* = 0.243), a clinically relevant trend but limited by small sample size. A higher age-adjusted Charlson Comorbidity Index (ACCI) was strongly associated with unsuccessful outcomes (median 5.5 vs 2, *p* = 0.001). Older age was also associated with lower success after stricture treatment (*p* = 0.012). These findings likely reflect surgical selection, as older and frailer patients were more often deemed unfit for reconstructive surgery rather than experiencing technical failure of surgery itself. The presence of an impacted stone at the time of URS was not associated with a higher chance of unsuccessful resolution compared to non-impacted stones (50% vs 22%), *p* = 0.141 in the present study.

## Discussion

In our cohort, the overall treatment success among patients who developed de novo ureteral strictures following URS appeared limited. Poorer outcomes were associated with higher comorbidity burden, and older patient age. Our findings complement one of the few other studies in this area. May et al. reported a North American series of 38 patients, in which 27.5% achieved resolution with endoscopic techniques alone, 37.5% underwent reconstructive surgery, and the remainder required lifelong drainage via nephrostomy or ureteral stent.^21^

Given the high volume of URS being performed worldwide, which is likely to continue to rise, our observations may hold increasing clinical relevance; however, they should be interpreted with caution as they are derived from a small cohort. Debate continues regarding the precise aetiology of de novo ureteral strictures developing after URS.^22^ A history of an impacted stone has been widely documented as a risk factor.^23,24^ In such cases, it can be difficult to determine how much ‘blame’ can be fairly attributed to ureteroscopic manipulation, and therefore considered iatrogenic, and to what extent stricture development was largely inevitable due to the degree of stone impaction. Rates of ureteral stricture(s) following URS have been reported to be higher among older adults. One possible explanation is that this demographic tends to present later after the onset of kidney stone symptoms, thereby increasing the likelihood of stone impaction.^25,26^ A recent international Delphi consensus study identified several risk factors for ureteral stricture formation following endoscopic treatment, including patient-related factors such as diabetes and prior radiotherapy, stone-related factors such as impaction, and intervention-related factors including the use of larger ureteroscopes and higher laser power settings.^27^

Many centres do not necessarily organise routine follow up for imaging. Indeed, in a statewide clinical registry study in Michigan, only 36.3% were documented to have undergone imaging follow up post URS.^28^ Reasons to perform follow up imaging are to not only determine stone free status but also rule out complications such as stricture disease. Optimal timing of follow up imaging also lacks consensus. In a cohort of 1066 patients imaged with CT at 1–2 months following URS, 58.6% of cases with new-onset hydronephrosis were found to be transient on later follow-up.^16^ This finding suggests that imaging follow-up at 3–6 months, as is conventional in many centres, may be more appropriate. However, later imaging schedules may delay the assessment of stone-free status in some patients. Therefore, a tailored approach to imaging follow-up appears most suitable, one that considers individual patient factors, the availability of local imaging resources, and the associated cost implications.^29^

Reported rates of stricture formation vary worldwide, and one reason is due to the lack of unique ICD code for stricture post endourological interventions. In centres where follow up imaging may not be routinely organised, or limited to only plain X ray, which would miss silent obstruction, the rates may be lower than reality. A total of 32.5% of the patients in our study had no symptoms from their strictures and would not have been recognised with such a follow-up. This figure is higher than previously reported in the literature.^16^

As well as laser technology, use of ureteral access sheaths (UAS) generates discussion regarding the associated risks of stricture formation.^30–33^ However, European Association of Urology (EAU) guidelines highlight there is no evidence to document that conventional UAS lead to a higher long term risk.^34^ On the other hand, in a previous study by Ulvik et al., the use of UAS was associated with a 4.6-fold increase in the risk of stricture development after URS lithotripsy.^35^ At the same time, with the advent and expanding use of suction sheath technologies, their potential to influence, if not heighten, the risk of stricture formation warrants further investigation.^36^

Novel methods to treat ureteral strictures have been studied and are under continued development. One example is dilatation with a ballon coated with paclitaxel and dextran.^37,38^ Using this method, Kallidonis et al., reported outcomes in a preliminary series of 25 patients with a success rate of 88% at 12 months reported.^39^ While they do not result in stricture resolution, self-expanding metallic ureteral stents such as the Memokath™ and Allium™ offer a management option for patients with benign ureteral obstruction who are stent-dependent, allowing for longer intervals between routine stent exchanges.^40,41^

### Limitations and strengths

This study has several limitations. First, it is a single-centre, retrospective analysis. Because our cohort also included patients who were referred after undergoing initial ureteroscopy (URS) at other centres, we were unable to estimate the overall incidence of de novo stricture formation following URS. The limited sample size reduces the statistical power of the analysis; accordingly, the observed associations should be viewed as exploratory only.

Despite these constraints, the present findings can help guide counselling of patients with newly diagnosed strictures and inform decisions regarding the likely efficacy of surgical interventions. This study also contributes to the very limited body of literature on post-URS ureteral strictures, which stands in clear contrast to the large number of URS procedures performed in real-world practice and is therefore extremely relevant. Future prospective studies with longer follow-up are needed to more comprehensively define the burden of this condition.

## Conclusion

In this retrospective cohort, fewer than half of patients with de novo ureteral strictures following URS achieved long-term resolution. Although the cohort size limits the strength of inference, the findings highlight the meaningful clinical burden of this complication and reinforce the importance of preventive strategies, appropriate follow-up imaging, and patient counselling. Further prospective studies are needed to validate these observations and refine management approaches.
